# Optimization of *in vitro* and *ex vitro Agrobacterium rhizogenes*-mediated hairy root transformation of soybean for visual screening of transformants using *RUBY*

**DOI:** 10.3389/fpls.2023.1207762

**Published:** 2023-07-07

**Authors:** Mohsen Niazian, François Belzile, Shaun J. Curtin, Maxime de Ronne, Davoud Torkamaneh

**Affiliations:** ^1^ Département de Phytologie, Université Laval, Québec City, QC, Canada; ^2^ Institut de Biologie Intégrative et des Systèmes (IBIS), Université Laval, Québec City, QC, Canada; ^3^ Centre de recherche et d’innovation sur les végétaux (CRIV), Université Laval, Québec City, QC, Canada; ^4^ Plant Science Research Unit, United States Department of Agriculture (USDA), St Paul, MN, United States; ^5^ Department of Agronomy and Plant Genetics, University of Minnesota, St. Paul, MN, United States; ^6^ Center for Plant Precision Genomics, University of Minnesota, St. Paul, MN, United States; ^7^ Center for Genome Engineering, University of Minnesota, St. Paul, MN, United States; ^8^ Institute Intelligence and Data (IID), Université Laval, Québec City, QC, Canada

**Keywords:** hairy root transformation, gene functional analysis, artificial neural networks, soybean, visual detection

## Abstract

*In vitro* and *ex vitro Agrobacterium rhizogenes*-mediated hairy root transformation (HRT) assays are key components of the plant biotechnology and functional genomics toolkit. In this report, both *in vitro* and *ex vitro* HRT were optimized in soybean using the *RUBY* reporter. Different parameters including *A. rhizogenes* strain, optical density of the bacterial cell culture (OD_600_), co-cultivation media, soybean genotype, explant age, and acetosyringone addition and concentration were evaluated. Overall, the *in vitro* assay was more efficient than the *ex vitro* assay in terms of the percentage of induction of hairy roots and transformed roots (expressing *RUBY*). Nonetheless, the *ex vitro* technique was deemed faster and a less complicated approach. The highest transformation of *RUBY* was observed on 7-d-old cotyledons of cv. Bert inoculated for 30 minutes with the R1000 resuspended in ¼ B5 medium to OD_600_ (0.3) and 150 µM of acetosyringone. The parameters of this assay also led to the highest percentage of *RUBY* through two-step *ex vitro* hairy root transformation. Finally, using machine learning-based modeling, optimal protocols for both assays were further defined. This study establishes efficient and reliable hairy root transformation protocols applicable for functional studies in soybean.

## 1 Introduction

A trio of biotechnologies (i.e., biomedicine, plant breeding and industrial) referred to as the new techno-economic paradigm has been proposed to help drive the world economy and mitigate climate change (Tylecote, 2019). Among them, green plant biotechnology has the potential to impact human life greatly by its influence on food production and security (Steinwand and Ronald, 2020). Biotechnological plant breeding is faster than conventional plant breeding for improving traits of interest (Tylecote, 2019). Recombinant DNA technology made it possible to incorporate exogenous DNA into crop genomes (Gosal and Wani, 2018) and has led to the precision breeding era through the development of custom-designed nucleases, zinc-finger nucleases (ZFNs) (Sander et al., 2007; Curtin et al., 2011), transcription activator-like effector nucleases (TALENs) (Cermak et al., 2011; Christian et al., 2012) and most recently clustered regulatory interspaced short palindromic repeats (CRISPR) (Čermák et al., 2017). *Agrobacterium*-mediated transformation (AMT) is the preferred biotechnological technique to deliver foreign DNA into plants (Ozyigit, 2020). Due to its Ti or Ri plasmids, both *A. tumefaciens* and *A. rhizogenes* (synonym = *Rhizobium rhizogenes*) can infect cells and transfer DNA (T-DNA) that integrates into the host genome (Bahramnejad et al., 2019). Confirmation of transgenes insertion into the plant genome is achieved by molecular biology techniques such as thermal asymmetric interlaced PCR (TAIL-PCR) (Singer and Burke, 2003), southern blotting (Sambrook et al., 1989), droplet digital PCR (ddPCR) (Collier et al., 2017) and more precisely through whole genome sequencing (WGS) (Curtin et al., 2018). Expression of antibiotic or herbicide resistance genes, known as selectable marker genes, is a common technique used for detection of transformed cells (Nishizawa-Yokoi et al., 2021). Finding the optimal selectable agent concentration used in conjunction with the selectable marker requires detailed experimentation to reach the effective inhibitory action of the selectable agent used, and to reduce the frequency of escape plants. These factors are considered essential for stable transformation of crop plants but can also be a bottleneck to use of selectable marker genes (Nyaboga et al., 2014).

Reporter genes that can be visually inspected is an alternative approach to identify transgenic plant material. The reporter genes β-glucuronidase (*GUS*) and luciferase (*LUC)* have been extensively used for screening transformants, however, they are destructive or require exogenous substrate and equipment for detection (Rakosy-Tican et al., 2007). The green fluorescent protein (*GFP*) is one of the most widely used reporter genes in gene transformations studies. It has no requirement for exogeneous substrates, is non-destructive and its expression is cell autonomous and independent of cell type and location (Zhang et al., 2001). Despite these benefits, *GFP* can induce deleterious effect on transformed cells and plant regeneration (Murray et al., 2004). Another fluorescence reporter gene with no detrimental effects on growth or fertility of transformed plants is *DsRed2* (Nishizawa et al., 2006). Detection of red fluorescent protein is much easier than *GFP*, because of the faint autofluorescence exhibited under red light (Sun et al., 2018). Zheng et al. (2020) compared the efficiency of screening using herbicide resistance (*basta*) and *GFP* fluorescence and reported that results of *GFP* fluorescence was more consistent with confirmed transgenics by the PCR approach. They have also showed that reporter genes are more reliable than selectable marker genes to identify true transgenic plants and to remove false positive events escaped from the antibiotic screening (Zheng et al., 2020). The last but most interesting group of reporter genes includes genes that are easily visible by naked eyes without the need for fluorescence or light imaging. These genes are mainly involved in production of colorful compounds such as anthocyanin (Kortstee et al., 2011; Khidr et al., 2017) or betalain (He et al., 2020) and their real-time *in vivo* detection is possible by naked eyes. *RUBY* is an artificial open reading frame of betalain, which can produce all the enzymes required for betalain biosynthesis, and its efficiency as visible reporter gene has been documented in both monocot and dicot plants (He et al., 2020).

Soybean (*Glycine max* (L.) Merr.) is one of the most economically important crops worldwide, providing human food, animal forage, vegetable oil and other industrial materials (such as natural tocopherols and sludge fatty acid) (Lyu et al., 2021). In addition, soybeans have a great ability to fix atmospheric nitrogen, with the help of specialized soil bacteria (Torkamaneh et al., 2020), which eliminates the need to apply nitrogen fertilizers, chemicals whose production and use contribute significantly to the greenhouse gas emissions. This makes soybean a very attractive crop from an environmental point of view. In the past few decades several candidate genes have been identified for different agronomic and physiological traits in soybean. The function of these candidate genes, such as *GmNARK*, *NFR5*, *SYMRK* and *GmSPX-RING1*, can be quickly and efficiently validated through AMT hairy root transformation (Cai et al., 2015). Confirmation of transgene insertion, followed by *A. rhizogenes* co-transformation, is an important step for the establishment of hairy roots with the gene of interest for various studies (Bahramnejad et al., 2019). A reporter gene directly visible by naked eyes in the early stages of hairy root initiation can greatly facilitate the detection of transformants and accelerate functional analysis (Niazian et al., 2022). In this study, we (i) optimized both an *in vitro* and an *ex vitro* hairy root transformation (HRT) assay for soybean using four *A. rhizogenes* strains and three soybean genotypes and (ii) determined whether *RUBY* can be observed in the early stages of both *in vitro* and *ex vitro* HRT assays and (iii) investigated its ability as reporter marker in different soybean genotypes with different genetic backgrounds.

## 2 Materials and methods

### 2.1 Plant materials

Three routinely transformed soybean cultivars, Williams82 (PI 518671), Jack (PI 540556), and Bert (PI 557010), along with a randomly selected line (20SS01), were used as plant materials to establish *in vitro* and *ex vitro* hairy root transformation protocols. All plant materials were obtained from seed collection of Université Laval and United States Department of Agriculture (USDA). Explants for the *in vitro* assay were obtained by germination of sterilized soybean seeds in culture vessels containing 50 ml of ¼ MS (Murashige and Skoog, 1962) medium (Taylor et al., 2006; Govindarajulu et al., 2008; Curtin et al., 2011). Seed were germinated in a growth chamber (Conviron Inc., MB, Canada) set for 16hr/26°C:8hr/22°C (light: dark) with 80% humidity.

For *ex vitro* experiments, soybean seedlings were obtained by germination of sterilized seeds in Jiffy^®^ seed starter peat pellets (Jiffy Group, Canada). Seeds were kept in a growth chamber at 28°C and irrigated daily with sterilized distilled water until germination. Then, regular irrigation of emerged seedlings was conducted using a mix of tap water supplemented with minerals outlined in **Table S1**.

### 2.2 Binary vector construct and *A. rhizogenes* transformation

The plasmid binary construct pARSCL504 [pTRANS_230] 35S:Ω:Ruby (Addgene # 198636) harboring the visually detectable *RUBY* reporter gene and the *bar* selectable marker was used in this study, hereafter known as the *RUBY* binary vector (RBV). The *RUBY* gene was originally sourced from Addgene (#160908) (He et al., 2020). An internal AarI site in the coding region was removed and cloned into pMC-6-CcdB-9 (Addgene #197812) and named pMC-6-Ruby-9 (Addgene #197740) (Chamness et al., 2022). Ruby was next assembled by golden-gate cloning into the pJC226 [pMOD_C] (Addgene #198637) along with plasmid components 35S promotor (pICH41373, Addgene #50252), the 5′-leader sequence of *Tobacco mosaic virus* (TMV) Ω (pICH41402, Addgene #50285) and the 35S poly A terminator (pICH41414, Addgene #50337) to generate pARSCL212 [pMOD_C] 35S:Ω:Ruby:t35S, (Addgene #198638) (Engler et al., 2014; Čermák et al., 2017; Chamness et al., 2022) (**Figure S1**). *A. rhizogenes* competent cells were prepared according to a freeze-thaw protocol (An et al., 1989; Chen et al., 1994) and these cells were transformed with the *RUBY* binary vector. Transformed colonies were selected on Luria-Bertani (LB) (Bertani, 1951) plates containing 50 mg/L kanamycin and glycerol stocks were prepared by suspension of fresh single colonies in liquid LB medium containing 50% (vol/vol) glycerol and kept at -80°C.

### 2.3 *In vitro* hairy root transformation

Glycerol stocks of *A. rhizogenes* strains harboring the *RUBY* binary vector were suspended in 150 ml of YEP liquid medium (10 g/L Bacto Peptone + 5 g/L yeast extract + 5 g/L NaCl, pH=7.0) (Lu and Kang, 2008) supplemented with 50 mg/L kanamycin and incubated at 28°C, 200 rpm for 48 hours. The cells were pelleted by centrifugation at 4°C, 6,000 rpm for 10 minutes re-suspended in MS or B5 (Gamborg et al., 1968) liquid media and adjusted to the desired cell density (OD_600_) (Chen et al., 2018). Centrifugation and re-suspension were repeated to remove traces of YEP media. *A. rhizogenes* suspensions were further incubated at 28°C, 200 rpm for 1 hour prior to inoculation. The cotyledons along with ~0.5cm hypocotyls were separated and submerged individually into the suspension (without agitation). Three *in vitro* experiments were conducted to assess the impact of different parameters on *A. rhizogenes*-mediated hairy root transformation in soybean as detailed below.

#### 2.3.1 *A. rhizogenes* parameters

The first experiment was conducted to assess the effect of three parameters (*A. rhizogenes* strain, bacterial cell concentration and inoculation duration) on the efficiency of hairy root induction and transformation on 7-d-old cotyledons of the 20SS01 genotype. This experiment was completely randomized with three replications (Petri dishes) and three factors for a total of 36 treatments (4 × 3 × 3); A: four *A. rhizogenes* strains (R1000, A4, ARqual and K599), B: three cell densities (OD_600_ = 0.3, 0.5 and 0.8) and C: three durations of inoculation (10, 20 and 30 min). After infection, five cotyledons were placed flat side up on sterile filter paper, prewetted with ¼ MS medium and co-cultivated in the dark (28°C) for three days. After infection the explants were then washed with sterile distilled water supplemented with cefotaxime (300 mg/L) for 30 min slow agitation to eradicate *Agrobacterium*. The explants were dried on sterile filter paper and placed in solid ¼ MS medium containing cefotaxime (300 mg/L) and phosphinothricin (ppt) (3 mg/Land incubated in a growth chamber (Conviron Inc., MB, Canada) at 16hr/26°C:8hr/22°C (light:dark) with 80% humidity. Hairy root induction percentage and transformation efficiency were calculated by visual inspection of *RUBY* on the twentieth day of the experiment using equations 1 and 2:


(1) $$HR\left(\%\right)=\frac{N_{hr}}{N_{T}}\;\times 100$$

(2) $$TE\left(\%\right)=\frac{N_{RUBY}}{N_{I}}\;\times 100$$

Where, HR is the percentage of hairy root induction, N_hr_ is the number of cotyledons/seedlings with hairy roots (lengths ≥ 1 cm), N_T_ is the total number of inoculated cotyledons/infected seedlings, TE is transformation efficiency, N_RUBY_ is the number of cotyledons/seedlings with at least one hairy root expressing the *RUBY* gene, and N_I_ is the number of inoculated cotyledons/infected seedlings with hairy roots (length ≥ 1 cm) (Melito et al., 2010; Su et al., 2022).

#### 2.3.2 Interaction between *A. rhizogenes* strains and plant parameters

In the second experiment, the interaction of the *A. rhizogenes* strain and plant genotype and explant age were evaluated. The cotyledons were prepared identically to experiment one with the following parameters: A: four soybean genotypes (Williams, Jack, Bert and 20SS01), B: four *A. rhizogenes* strains (R1000, A4, ARqual and K599) and C: three ages of explants (5-, 7- and 10-d-old cotyledons). As in the previous experiment, the *A. rhizogenes* was eradicated following co-cultivation using the method described above. Hairy root induction percentage and efficiency were calculated by visual inspection of *RUBY* on the twentieth day of the experiment using equations 1 and 2.

#### 2.3.3 *In vitro* culture parameters

In the third experiment, the effect of co-cultivation media and the concentration of acetosyringone was evaluated on 7-d-old cotyledon explants of cv. Bert. The cotyledons were prepared identically as described above with the following parameters: A: three concentrations of acetosyringone (100, 150 and 200 µmol/L) and B: four basal root induction media (B5, ¼ B5, MS and ¼ MS). Inoculated explants (five cotyledons per Petri dishes) were transferred to Petri dishes containing sterile filter paper and ¼ MS medium and supplemented with different concentrations of acetosyringone. As in the previous experiment, the *A. rhizogenes* was eradicated following co-cultivation using the method described above. Hairy root induction and transformation efficiency were calculated after 20 days incubation of culture vessels in the growth chamber with the same conditions as mentioned above, using equations 1 and 2.

### 2.4 *Ex vitro* hairy root transformation

The *ex vitro* HRT assay was evaluated by comparing the *A. rhizogenes* strains and soybean genotype. Seedlings of four soybean genotypes were inoculated with four different *A. rhizogenes strains*. The experiment was carried out with three replications and three factors for a total of 48 treatments (4 × 4 × 3): A: four *A. rhizogenes* strains (R1000, A4, ARqual and K599), B: four soybean genotypes (Williams, Jack, Bert and 20SS01) and C: three ages of explants (5-, 7- and 10-d-old seedlings). Single colonies of *Agrobacterium*, harboring the *RUBY* binary vector, were suspended in 1 ml liquid LB medium containing 15% (vol/vol) glycerol and 200 µL was spread onto the solid YEP plates with 50 mg/L kanamycin. Following overnight incubation at 28°C, the dense bacterial lawn was used infect soybean seedling scions as described by Kereszt et al. (2007). After inoculation the seedlings were incubated in a humid chamber and irrigated with nutrient solution (**Table S1**). Hairy root induction percentage and efficiency were calculated by visual inspection of *RUBY* on the twentieth day of the experiment using equations 1 and 2.

### 2.5 Molecular evaluation of putative transgenic hairy roots and expression pattern of the *RUBY* gene

All putative transgenic hairy roots (red roots) were evaluated for the presence of the T-DNA by PCR using genomic DNA as template and primers that amplify the *bar* gene (F: 5’-GACAAGCACGGTCAACTTCC-3’; R: 5’-AGTCCAGCTGCCAGAAACC-3’). The *A. rhizogenes* contamination was checked by PCR using *rol* C specific primers (**Table S2**) (Thwe et al., 2016).

The expression of *RUBY* at different developmental stages of hairy root production was evaluated by real-time quantitative PCR (RT-qPCR). RNA was extracted using TRIzol^®^ reagent (Thermo Fisher Scientific) and purified by RNeasy Plant Mini Kit (Qiagen). First strand synthesis was carried out using the iScript Advanced cDNA Synthesis Kit for RT-qPCR (Bio-Rad, Canada). RT-qPCR was conducted on a 7500 Fast Real-time PCR system (Applied Biosystems) using the iQ SYBR green supermix (Bio-Rad, Canada) with synthesized cDNA as template and *RUBY*-specific primers designed using the Primer3 web-based software (https://bioinfo.ut.ee/primer3-0.4.0/) (**Table S2**). The 2^−ΔΔCt^ method (Livak and Schmittgen, 2001) was used to calculate the relative expression level of the *RUBY* gene in samples, with three biological replicates, taken from three different parts of red roots (**Figure S2**). A putative ubiquitin gene (Gma.441.1.S1_at) and a putative actin gene (GmaAffx.90181.1.A1_at) were used as housekeeping genes (**Table S2**) (Wang et al., 2010; Wang et al., 2012).

### 2.6 Modeling and optimization of *in vitro* and *ex vitro A. rhizogenes*-mediated hairy root transformation

An artificial neural network (ANN) and a genetic algorithm (GA) were applied to predict-optimize both *in vitro* and *ex vitro A. rhizogenes*-mediated hairy root transformation of soybean. The multilayer perceptron (MLP) of the ANN (Hesami et al., 2019) was used to predict the dependent outputs using the independent input variables. *A. rhizogenes* strain, cell density, inoculation duration, plant genotype, age of explants, concentration of acetosyringone and type of basal root induction medium were the inputs of the established model to predict the percentage of hairy root induction and transformation efficiency of *RUBY* in *in vitro* experiments. *A. rhizogenes* strain, plant genotype and age of explants were the inputs of the established model for *ex vitro* experiments. The best topology of both *in vitro* and *ex vitro* models (optimal number of hidden units and the number of neurons in each node) were determined using a trial and error-based approach (Hesami and Jones, 2020). Linear function (purelin) and hyperbolic tangent sigmoid function (tansig) were used as transfer functions of the output and hidden layers, respectively. The Levenberg-Marquardt (LM) algorithm was applied as a learning algorithm for adjusting bias and weights (Niazian et al., 2019). Total sample data sets (for both *in vitro* and *ex vitro* experiments) were partitioned into two subsets for training (75%) and testing (25%) (Niazian et al., 2019). The performance of ANN-MLP models was assessed through coefficient of determination (R^2^), mean bias error (MBE), and root mean square error (RMSE), based on the following equations:


(3) $$R^{2}=1-\frac{\Sigma_{\text{i}=1}^{n}{\left(y_{i}-{\hat{y}}_{i}\right)}^{2}}{\Sigma_{\text{i}=1}^{n}{\left(y_{i}-{\hat{y}}_{i}\right)}^{2}}$$

(4) $$RMSE=\sqrt{(\sum_{i=1}^{n}{\left(y_{i}-{\hat{y}}_{i}\right)}^{2})/n}$$

(5) $$MBE=\frac{1}{n}\;\sum_{i=1}^{n}\left(y_{i}-{\hat{y}}_{i}\right)$$

where $y_{i}$ is the value of prediction, n is the number of data, and ${\hat{y}}_{i}$ is the value of observation (Pepe et al., 2021).

The optimum levels of input values to maximize each fitness function (predicted percentage of hairy root induction and expression of *RUBY*) were determined by GA evolutionary optimization algorithm. For this purpose, an initial population of 200, generation number of 1000, mutation rate of 0.05, uniform mutation function, Roulette Wheel selection function, cross-over fraction of 0.6 and Two-point cross- over function were considered to complete the optimization process by satisfying one of the termination criteria (Pepe et al., 2021).

### 2.7 Statistical analyses

Three replications, each with five explants, were employed for each treatment (combination of parameters) in the above mentioned *in vitro* and *ex vitro* experiments.

Analysis of variance (ANOVA) and means comparison analysis were carried out using SAS^®^ (SAS Institute Inc., Cary, NC). The normal distribution of data was checked before the analysis of variance. Means were compared using Duncan’s multiple range test (DMRT) at 5% (P ≤ 0.05) probability level. MATLAB software (MathWorks 2022a) was used for the hybrid MLP-GA analyses. All graphs were generated using R software (version 4.2.1)

## 3 Results

### 3.1 Hairy root induction and *RUBY* expression via the *in vitro* method

**Figure 1:**
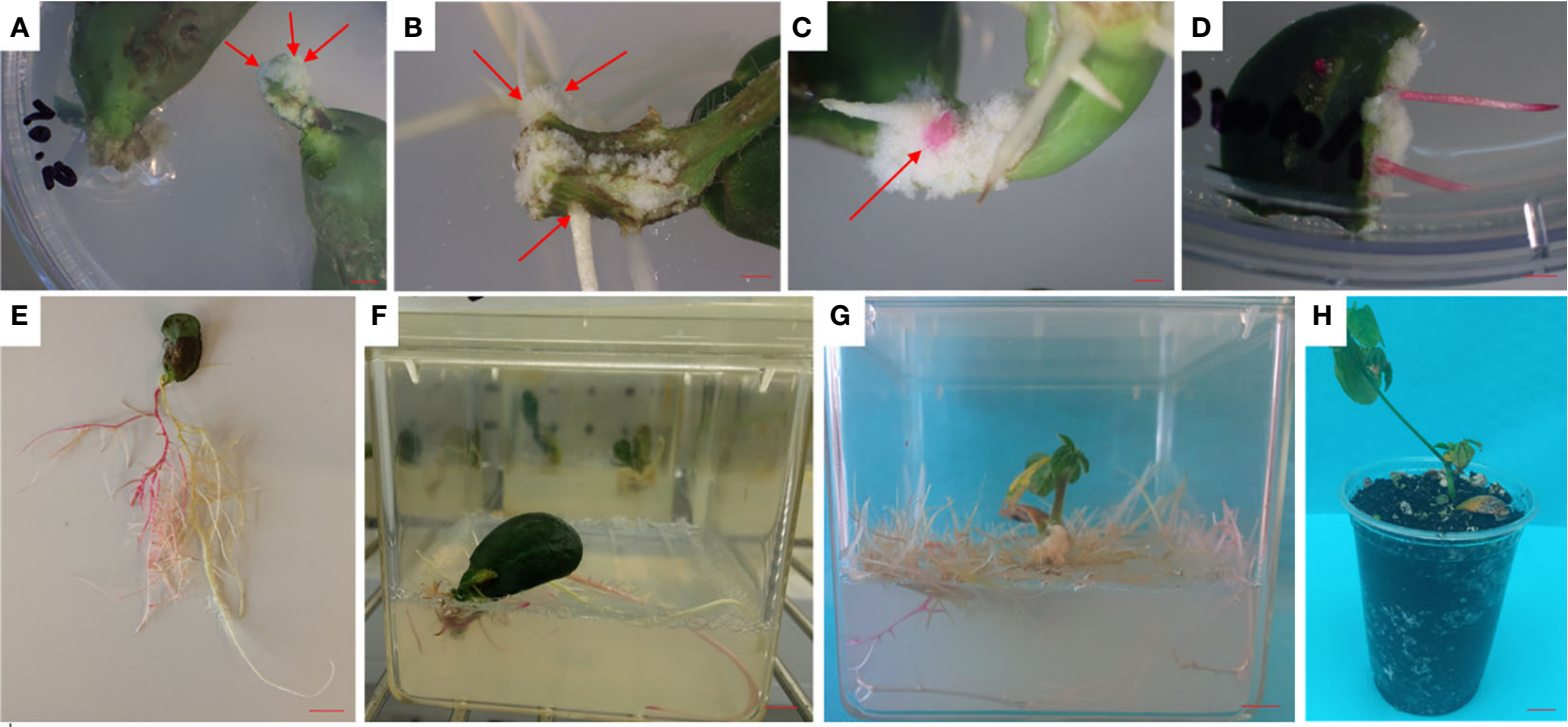
*In vitro* hairy root induction and *RUBY* expression in soybean. **(A)** Emergence of white calli at the basal end of cultured cotyledons (red arrows) (bar= 5 mm). **(B)** The emergence of the first hairy roots on the surface of elongated hypocotyls (red arrows) (bar= 5 mm). **(C)** The first signs of red hairy roots on the surface of induced calli (red arrow) (bar= 5 mm). **(D)** Induced red hairy roots from accidentally broken cotyledons (bar= 5 mm). **(E)** Elongation of red hairy roots in root induction medium (bar= 3 cm). **(F)** Shoot induction from cotyledons with red hairy roots (bar= 3 cm). **(G)** Composite soybean plantlet with a large mass of red hairy roots expressing *RUBY* (bar= 3 cm). **(H)** Acclimatized composite soybean plant (bar= 3 cm).

After seven days explants inoculated with *A. rhizogenes* produced white calli at the base of the cotyledons (**Figure 1A**). In the next seven days, hypocotyl elongation and emergence of the hairy roots could be seen (**Figure 1B**). On the fifteenth day, the first signs of transformed roots were observed on the white calli (**Figure 1C**). Ruby-colored hairy roots observed from accidentally broken cotyledons (**Figure 1D**). Long red hairy roots, expressing *RUBY* 20 days after inoculation (**Figure 1E**). Root induction from inoculated with ¼ MS media containing 500 mg/L carbenicillin (**Figure 1F**) and composite plantlets (containing wild-type shoots with transgenic roots) with a large mass of red hairy roots were obtained after a further seven days (**Figure 1G**). Acclimatized composite plants (**Figure 1H**).

### 3.2 Hairy root induction and *RUBY* expression via the *ex vitro* method

**Figure 2:**
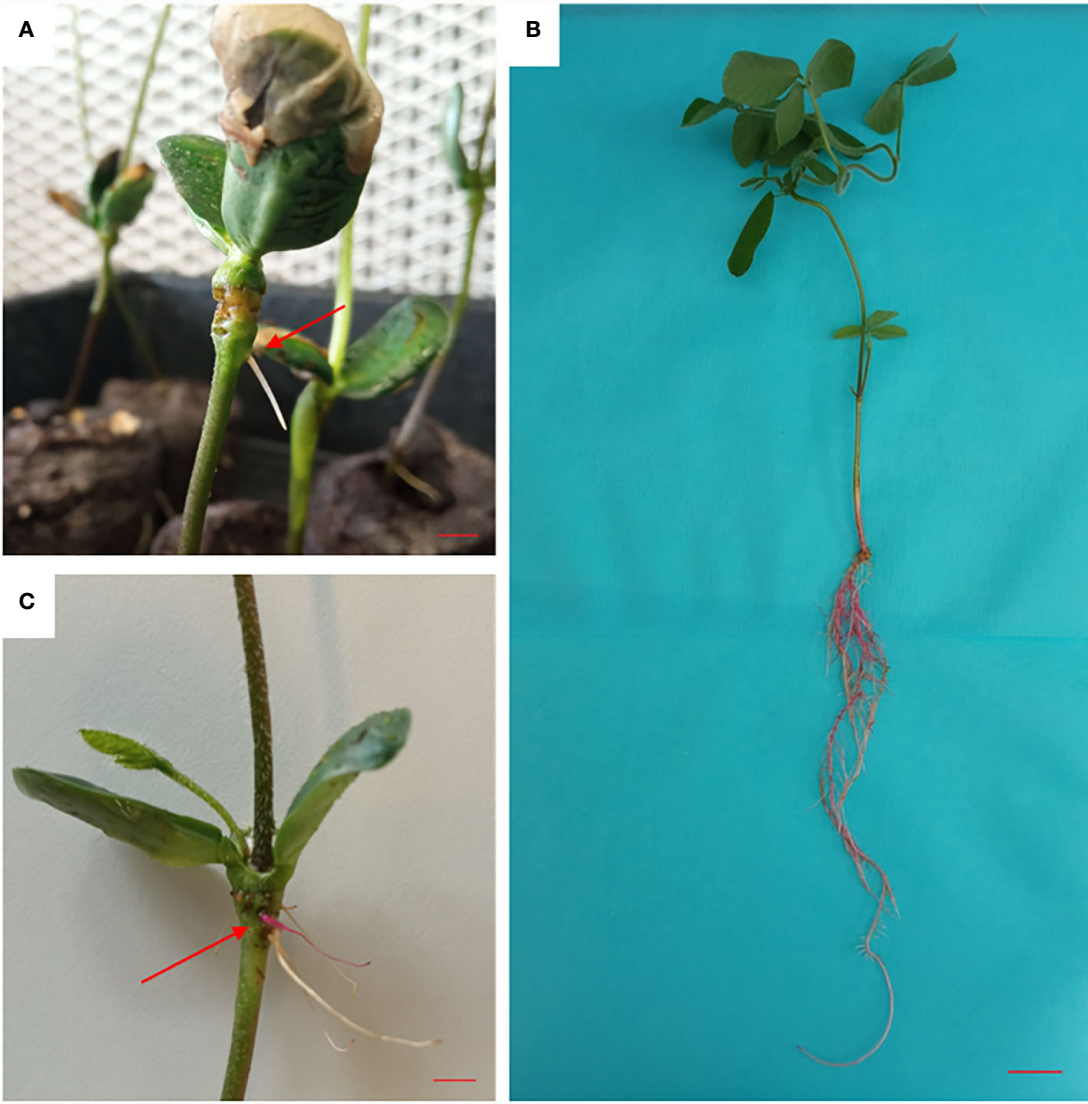
Hairy root induction and *RUBY* gene expression in soybean using the hypocotyl stabbing technique. **(A)** Emergence of the first hairy roots at the wounding/infection sites of hypocotyls (red arrow) (bar= 1 cm). **(B)** Emergence of red hairy roots, *RUBY* expression at the infection sites of hypocotyls (red arrow) (bar= 1 cm). **(C)** Composite soybean plants with fully red hairy roots (bar= 3 cm).

Infected plantlets were kept in a humid chamber and regularly sprayed with sterilized distilled water (**Figure S3A**). At the point of wounding of the hypocotyls, the first hairy roots were observed on the fourteenth day of the experiment (**Figure 2A**). Red hairy roots, expressing the *RUBY* gene, emerged along with untransformed roots (**Figure 2B**). On the twentieth day of the experiment, when the hairy roots reached a length of about 5 cm (**Figure S3B**), the primary roots were removed by cutting the hypocotyl under the emergence point of hairy roots. As plants were grown in peat pellets, it was relatively easy to handle each individual plant (**Figure S3C**). Non-transformed white hairy roots were removed, and composite soybean plants were transplanted to pots (10 × 10 cm) filled with turface and perlite and irrigated with nutrient solution. Composite plantlets with a large mass of red hairy roots were obtained after one week (**Figure 2C**).

### 3.3 *In vitro* optimization experiments

#### 3.3.1 Optimization of *A. rhizogenes* parameters

The ANOVA analysis showed that the effects of *A. rhizogenes* strain, cell culture density and duration of inoculation on the percentage of hairy root induction and transformation efficiency were significant (*p* ≤ 0.01) (**Table S3**). As shown in **Table 1**, the comparison of means revealed that the R1000 and A4 strains were the most and least efficient for hairy root induction, respectively. For A4, ARqual and K599 strains, the highest mean percentages of hairy root induction were achieved at an OD_600_ = 0.5, whereas the best cell density for the R1000 strain was OD_600_ = 0.3. Explants inoculated for 30 min responded most favorably, resulting in the highest induction of hairy roots across all strains and cell densities. The highest mean percentage of hairy root induction (100%) was achieved by two of the 36 treatments (R1000/OD_600_ = 0.3/30 min and K599/OD_600_ = 0.5/30 min).

**Table 1:** Mean percentage of hairy root induction in soybean line 20SS01 via *in vitro* transformation.

Strain	Cell Density (OD_600_)	Inoculation duration (min)		
		10	20	30
R1000	0.30	40.00 ± 2.98^e^	73.33 ± 2.98^b^	100 ± 0.00^a^
	0.50	53.33 ± 2.98^d^	60.00 ± 2.98^cd^	66.66 ± 2.98^bc^
	0.80	0.00 ± 0.00^h^	6.66 ± 2.98^gh^	20.00 ± 0.00^fg^
A4	0.30	0.00 ± 0.00^h^	0.00 ± 0.00^h^	6.66 ± 2.98^gh^
	0.50	13.33 ± 2.98^fh^	20.00 ± 2.98^fg^	40 ± 0.00^e^
	0.80	6.66 ± 2.98^gh^	13.33 ± 2.98^fh^	6.66 ± 2.98^gh^
ARqual	0.30	0.00 ± 0.00^h^	0.00 ± 0.00^h^	20.00 ± 0.00^fg^
	0.50	20.00 ± 0.00^fg^	26.66 ± 2.98^f^	60.00 ± 0.00^cd^
	0.80	20.00 ± 0.00^fg^	26.66 ± 2.98^f^	40.00 ± 0.00^e^
K599	0.30	0.00 ± 0.00^h^	6.66 ± 2.98^gh^	20.00 ± 0.00^fg^
	0.50	13.33 ± 2.98^fh^	66.66 ± 2.98^bc^	100 ± 0.00^a^
	0.80	6.66 ± 2.98^gh^	20.00 ± 0.00^fg^	26.66 ± 2.98^f^

Values represent the mean ± standard error from three replications. Values followed by the same letter are not significantly different at P< 0.05. Three parameters (strain, cell density and duration of inoculation) were explored in a factorial experiment where each of 36 treatments (five cotyledons/treatment) was replicated three times.

Transformation efficiency was extremely variable depending on the strain used (**Table 2**). Indeed, no transgenic roots were obtained at all (total of 9 treatments) with the A4 strain while R1000 was the most efficient. While OD_600_ = 0.5 was the most efficient cell density with two strains (ARqual and K599), OD_600_ = 0.3 was the most efficient for the R1000 strain. Overall, we found that 30 min was the best duration of inoculation as it led to the highest transformation efficiencies across the R1000, ARqual and K599 strains. The highest transformation efficiency (66.66%) was obtained with the treatment R1000/OD_600_ = 0.3/30 min, followed by K599/OD_600_ = 0.5/30 min (53.33%).

**Table 2:** Mean percentage of transformation efficiency in 20SS01 genotype via *in vitro* transformation.

Strain	Cell density (OD_600_)	Inoculation duration (min)		
		10	20	30
R1000	0.30	0.00 ± 0.00^e^	20.00 ± 5.16^cd^	66.66 ± 2.98^a^
	0.50	13.33 ± 2.98^de^	0.00 ± 0.00^f^	26.66 ± 2.98^c^
	0.80	0.00 ± 0.00^f^	0.00 ± 0.00^f^	0.00 ± 0.00^f^
A4	0.30	0.00 ± 0.00^f^	0.00 ± 0.00^f^	0.00 ± 0.00^f^
	0.50	0.00 ± 0.00^f^	0.00 ± 0.00^f^	0.00 ± 0.00^f^
	0.80	0.00 ± 0.00^f^	0.00 ± 0.00^f^	0.00 ± 0.00^f^
ARqual	0.30	0.00 ± 0.00^f^	0.00 ± 0.00^f^	0.00 ± 0.00^f^
	0.50	0.00 ± 0.00^f^	0.00 ± 0.00^f^	13.33 ± 2.98^de^
	0.80	0.00 ± 0.00^f^	0.00 ± 0.00^f^	6.66 ± 2.98^ef^
K599	0.30	0.00 ± 0.00^f^	0.00 ± 0.00^f^	0.00 ± 0.00^f^
	0.50	0.00 ± 0.00^f^	13.33 ± 2.98^ef^	53.33 ± 2.98^b^
	0.80	0.00 ± 0.00^f^	0.00 ± 0.00^f^	0.00 ± 0.00^f^

Values represent the mean ± standard error from three replications. Values followed by the same letter are not significantly different at P< 0.05. Three parameters (strain, cell density and duration of inoculation) were explored in a factorial experiment where each of 36 treatments (five cotyledons/treatment) was replicated three times.

#### 3.3.2 Interaction of *A. rhizogenes* and plant parameters

The main, two- and three-way interactions of plant genotype, strain, and explant age were highly significant (*p*<0.01) on hairy root induction and transformation efficiency (**Table S4**). Depending on the treatment, hairy root induction varied from 0 to 100% (**Table 3**). Bert and the 20SS01 genotype responded the best to the *in vitro* hairy root induction. The R1000 strain proved the most efficient while A4 was the least. Seven-d-old explants were more efficient than both 5-d and 10-d-old explants. The highest hairy root induction percentages (100%) were obtained by inoculation of 7-d-old cotyledons of four combinations of genotype and strain: Williams82 with strain K599, Bert with R1000, and the 20SS01 genotype with either R1000 or K599.

**Table 3:** Mean percentage of hairy root induction in soybean via *in vitro* transformation.

Plant genotype	Strain	Age of explants		
		5-d	7-d	10-d
Williams	R1000	46.66 ± 2.98^def^	73.33 ± 2.98^bc^	60.00 ± 0.00^cd^
	A4	0.00 ± 0.00^k^	6.66 ± 2.98^jk^	0.00 ± 0.00^k^
	ARqual	0.00 ± 0.00^k^	13.33 ± 2.98^ijk^	6.66 ± 2.98^jk^
	K599	20.00 ± 0.00^hij^	100.00 ± 0.00^a^	73.33 ± 2.98^bc^
Jack	R1000	13.33 ± 2.98^ijk^	46.66 ± 2.98^def^	26.66 ± 2.98^ghi^
	A4	0.00 ± 0.00^k^	13.33 ± 2.98^ijk^	6.66 ± 2.98^jk^
	ARqual	0.00 ± 0.00^k^	40.00 ± 0.00^efg^	13.33 ± 2.98^ijk^
	K599	13.33 ± 2.98^ijk^	60.00 ± 0.00^cd^	26.66 ± 2.98^ghi^
Bert	R1000	33.33 ± 2.98^fgh^	100.00 ± 0.00^a^	73.33 ± 2.98^bc^
	A4	0.00 ± 0.00^k^	13.33 ± 2.98^ijk^	6.66 ± 2.98^jk^
	ARqual	0.00 ± 0.00^k^	33.33 ± 2.98^fgh^	6.66 ± 2.98^jk^
	K599	13.33 ± 2.98^ijk^	80.00 ± 0.00^b^	53.33 ± 2.98^de^
20SS01	R1000	26.66 ± 2.98^ghi^	100.00 ± 0.00^a^	53.33 ± 2.98^de^
	A4	0.00 ± 0.00^k^	40.00 ± 0.00^efg^	26.66 ± 2.98^ghi^
	ARqual	0.00 ± 0.00^k^	60.00 ± 0.00^cd^	20.00 ± 5.16^hij^
	K599	6.66 ± 2.98^jk^	100.00 ± 0.00^a^	26.66 ± 2.98^ghi^

Values represent the mean ± standard error from three replications. Values followed by the same letter are not significantly different at P< 0.05. Three parameters (strain, plant genotype and explant age) were explored in a factorial experiment where each of 48 treatments (five cotyledons/treatment) was replicated three times.

Bert and Jack showed the highest and lowest percentages of *in vitro* transformation, respectively (**Table 4**). R1000 was the best strain and led to the highest transformation efficiency in all investigated soybean genotypes, however there was no significant difference between strains R1000 and K599 in Williams82 and Jack. Cotyledons at the age of 7-d responded most favorably, resulting in the highest transformation in 15 of the 16 treatments (strain × cultivar). The highest mean percentage of transformation (73.33%) was obtained with the combination of cv. Bert/R1000/7-d old explants.

**Table 4:** Mean percentage of transformation efficiency in soybean via *in vitro* transformation.

Plant genotype	Strain	Age of explants		
		5-d	7-d	10-d
Williams	R1000	13.33 ± 5.96^de^	53.33 ± 2.98^bc^	13.33 ± 2.98^de^
	A4	0.00 ± 0.00^e^	0.00 ± 0.00^e^	0.00 ± 0.00^e^
	ARqual	0.00 ± 0.00^e^	0.00 ± 0.00^e^	0.00 ± 0.00^e^
	K599	0.00 ± 0.00^e^	46.66 ± 2.98^c^	40.00 ± 5.16^c^
Jack	R1000	0.00 ± 0.00^e^	06.66 ± 2.98^de^	0.00 ± 0.00^e^
	A4	0.00 ± 0.00^e^	0.00 ± 0.00^e^	0.00 ± 0.00^e^
	ARqual	0.00 ± 0.00^e^	0.00 ± 0.00^e^	0.00 ± 0.00^e^
	K599	0.00 ± 0.00^e^	13.33 ± 2.98^de^	0.00 ± 0.00^e^
Bert	R1000	0.00 ± 0.00^e^	73.33 ± 2.98^a^	40.00 ± 5.16^c^
	A4	0.00 ± 0.00^e^	0.00 ± 0.00^e^	0.00 ± 0.00^e^
	ARqual	0.00 ± 0.00^e^	0.00 ± 0.00^e^	0.00 ± 0.00^e^
	K599	0.00 ± 0.00^e^	53.33 ± 2.98^bc^	20.00 ± 5.16^d^
20SS01	R1000	0.00 ± 0.00^e^	66.66 ± 2.98^ab^	13.33 ± 2.98^de^
	A4	0.00 ± 0.00^e^	0.00 ± 0.00^e^	0.00 ± 0.00^e^
	ARqual	0.00 ± 0.00^e^	13.33 ± 2.98^de^	0.00 ± 0.00^e^
	K599	0.00 ± 0.00^e^	46.66 ± 2.98^c^	0.00 ± 0.00^e^

Values represent the mean ± standard error from three replications. Values followed by the same letter are not significantly different at P< 0.05. Three parameters (strain, plant genotype and explant age) were explored in a factorial experiment where each of 48 treatments (five cotyledons/treatment) was replicated three times.

#### 3.3.3 The effects of root induction media and acetosyringone

**Figure 3:**
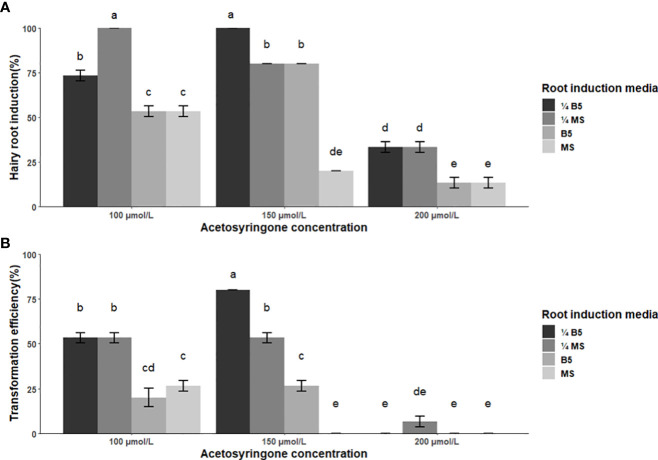
Effect of co-cultivation media and acetosyringone concentration on the efficiency of hairy root induction and transformation in soybean cv. Bert using *A rhizogene* strain R1000. **(A)** Percentage of explants showing hairy root induction under three acetosyringone concentrations and on four co-cultivation media. **(B)** Percent of transformed roots obtained using four root induction media and three acetosyringone concentrations. Values represent the mean ± standard error from three replicate trials. Treatments resulting in statistically different means (*p*< 0.05) are indicated by different letters.

Results of ANOVA revealed that the main and two-way interaction of acetosyringone concentration and root induction media were highly significant on both hairy root induction percentage and transformation efficiency (**Table S5**). At 150 µmol/L, acetosyringone proved the most efficient, thus resulting in the highest induction of hairy roots in 11 of the 12 treatments, while 200 µmol/L was the least. As for root induction media, ¼ MS and ¼ B5 media were the most efficient. Across all treatments, two produced hairy roots in 100% of the infected explants (100 µmol/L acetosyringone in ¼ MS medium and 150 µmol/L acetosyringone in ¼ B5 medium) (**Figure 3A**). Transformation efficiency varied from 0 to 80%. Acetosyringone at the concentration of 150 µmol/L caused the highest transformation in ¼ B5, ¼ MS and 1x B5 media, while a higher concentration (200 µmol/L) led to a significant decrease in the percentage of transformation. Overall, the highest transformation efficiency (80%) was obtained by application of 150 µmol/L acetosyringone in ¼ B5 medium (**Figure 3B**).

### 3.4 *Ex vitro* optimization

The main, two- and three-way interactions effect of strain, plant genotype and seedling age were significant on both hairy root induction and transformation efficiency at the 1% probability level, based on the results of ANOVA (**Table S6**). Overall, hairy root induction varied widely (from 0 to 100%) depending on the treatments (**Table 5**). 7-d-old seedlings responded most favorably, resulting in the highest induction of hairy roots in 15 of the 16 treatments (strain × cultivar). The R1000 strain proved the most efficient while A4 was the least. Finally, while all four genotypes proved proficient at producing hairy roots, cv. Bert and line 20SS01 generally responded the best to the *ex-vitro* hairy root induction. Across all treatments, two produced hairy roots in 100% of the infected seedlings (strains R1000 or K599 on 7-d-old seedlings of cv. Bert).

**Table 5:** Effect of *Agrobacterium rhizogenes* strain, plant genotype and age of seedling on the percentage of hairy root induction in the *ex vitro* method.

Strain	Plant genotype	Age of seedlings		
		5-d	7-d	10-d
R1000	Williams	46.66 ± 2.98^cdef^	60.00 ± 0.00^bcd^	93.33 ± 2.98^a^
	Jack	20.00 ± 0.00^ghi^	66.66 ± 2.98^bc^	33.33 ± 5.96^efgh^
	Bert	33.33 ± 2.98^efgh^	100.00 ± 0.00^a^	60.00 ± 5.16^bcd^
	20SS01	26.66 ± 5.96^fgh^	80.00 ± 5.16^ab^	46.66 ± 2.98^cdef^
A4	Williams	0.00 ± 0.00^i^	53.33 ± 2.98^cde^	13.33 ± 2.98^hi^
	Jack	0.00 ± 0.00^i^	66.66 ± 2.98^bc^	20.00 ± 5.16^ghi^
	Bert	13.33 ± 2.98^hi^	60.00 ± 0.00^bcd^	33.33 ± 2.98^efgh^
	20SS01	40.00 ± 0.00^defg^	93.33 ± 2.98^a^	33.33 ± 5.96^efgh^
ARqual	Williams	26.66 ± 8.94^fgh^	80.00 ± 0.00^ab^	40.00 ± 0.00^defg^
	Jack	20.00 ± 5.16^ghi^	60.00 ± 0.00^bcd^	20.00 ± 0.00^ghi^
	Bert	60.00 ± 5.16^bcd^	80.00 ± 0.00^ab^	33.33 ± 2.98^efgh^
	20SS01	26.66 ± 2.98^fgh^	60.00 ± 0.00^bcd^	40.00 ± 0.00^defg^
K599	Williams	33.33 ± 2.98^efgh^	60.00 ± 0.00^bcd^	40.00 ± 0.00^defg^
	Jack	20.00 ± 0.00^ghi^	60.00 ± 0.00^bcd^	26.66 ± 2.98^fgh^
	Bert	40.00 ± 0.00^defg^	100.00 ± 0.00^a^	40.00 ± 0.00^defg^
	20SS01	53.33 ± 5.96^cde^	93.33 ± 2.98^a^	53.33 ± 2.98^cde^

Values represent the mean ± standard error from three replications. Values followed by the same letter are not significantly different at P< 0.05. A total of 48 treatments were tested in replicated trials.

Transformation efficiency varied from 0 to 60%, depending on the treatments (**Table 6**). Seven-d-old seedlings responded most favorably, resulting in the highest transformation efficiency across all strains and genotypes (strain × genotype). The R1000 and A4 strains proved the most and least efficient, respectively. Cultivar Bert followed by line 20SS01generally responded the best to *ex vitro* transformation. Strain R1000 on 7-d explants of cv. Bert produced the highest transformation efficiency (60%) across all treatments.

**Table 6:** Effect of *Agrobacterium rhizogenes* strain, plant genotype and age of seedling on the percentage of transformation efficiency in the *ex vitro* method.

Strain	Plant genotype	Age of seedlings		
		5-d	7-d	10-d
R1000	Williams	0.00 ± 0.00^e^	46.66 ± 2.98^bc^	6.66 ± 2.98^gh^
	Jack	0.00 ± 0.00^h^	26.66 ± 2.98^def^	6.66 ± 2.98^gh^
	Bert	26.66 ± 2.98^def^	60.00 ± 0.00^a^	13.33 ± 2.98^fgh^
	20SS01	6.66 ± 2.98^gh^	53.33 ± 2.98^ab^	6.66 ± 2.98^gh^
A4	Williams	0.00 ± 0.00^h^	0.00 ± 0.00^h^	0.00 ± 0.00^h^
	Jack	0.00 ± 0.00^h^	0.00 ± 0.00^h^	0.00 ± 0.00^h^
	Bert	0.00 ± 0.00^h^	20.00 ± 0.00^efg^	0.00 ± 0.00^h^
	20SS01	0.00 ± 0.00^h^	6.66 ± 2.98^gh^	0.00 ± 0.00^h^
ARqual	Williams	0.00 ± 0.00^h^	20.00 ± 0.00^efg^	0.00 ± 0.00^h^
	Jack	0.00 ± 0.00^h^	20.00 ± 0.00^efg^	0.00 ± 0.00^h^
	Bert	0.00 ± 0.00^h^	40.00 ± 0.00^cd^	6.66 ± 2.98^gh^
	20SS01	0.00 ± 0.00^h^	33.33 ± 2.98^cde^	6.66 ± 2.98^gh^
K599	Williams	6.66 ± 2.98^gh^	33.33 ± 2.98^cde^	13.33 ± 2.98^fgh^
	Jack	0.00 ± 0.00^h^	20.00 ± 0.00^efg^	0.00 ± 0.00^h^
	Bert	0.00 ± 0.00^h^	53.33 ± 2.98^ab^	26.66 ± 2.98^def^
	20SS01	0.00 ± 0.00^h^	33.33 ± 2.98^cde^	6.66 ± 2.98^gh^

Values represent the mean ± standard error from three replications. Values followed by the same letter are not significantly different at P< 0.05. A total of 48 treatments were tested in replicated trials.

### 3.5 Machine learning-mediated development of an optimized protocol for hairy root transformation

The highest R^2^ values in both training and testing stages were obtained for hairy root induction percentage in the *in vitro* experiments (R^2^ = 0.94, 0.92), whereas the lowest R^2^ value was obtained in testing stage of the MLP model to predict the *RUBY* binary vector transformation in the *ex vitro* experiments (R^2^ = 0.82) (**Table 7**). The least amounts of RMSE and MBE performance indices were also achieved using MLP model to predict hairy root induction and *RUBY* binary vector transformation in the *in vitro* experiments (**Table 7**).

**Table 7:** Performance indices of applied MLP machine learning algorithm for modeling and predicting hairy root transformation of soybean in the *in vitro* and *ex vitro* experiments.

Experiment	Output	Performance index					
		R^2^		RMSE		MBE	
		Training	Testing	Training	Testing	Training	Testing
*In vitro*	Hairy root induction	0.94	0.92	7.16	12.29	-0.54	-1.41
	*RUBY* gene transformation	0.92	0.90	6.59	10.55	0.38	-0.94
*Ex vitro*	Hairy root induction	0.88	0.84	9.79	19.76	0.10	0.57
	*RUBY* gene transformation	0.88	0.82	6.06	10.20	-0.12	-1.02

MBE, mean bias error; R^2^, Coefficient of determination; RMSE, root mean square error.

**Figure 4:**
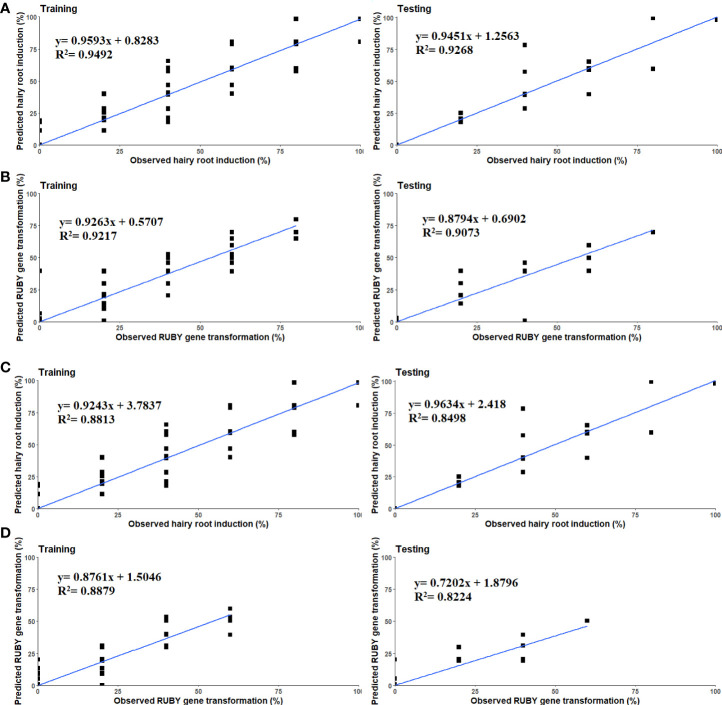
Scatter plot of experimental and predicted data of **(A, B)** Hairy root induction and *RUBY* binary vector transformation in the *in vitro*, **(C, D)** Hairy root induction and *RUBY* binary vector transformation in the *ex vitro* assay using multilayer perceptron in training and testing subsets.

The scatter plot of measured and predicted values of hairy root induction and *RUBY* gene transformation in ANN models showed a good fit correlation between experimental predicted data for both *in vitro* and *ex vitro* experiments (**Figures 4A–D**).

The results of GA evolutionary optimization algorithm predicted that inoculation of 6.89-d-old cotyledons of line 20SS01with R1000 strain at OD_600_ = 0.38 for 28.83 min, then incubation of infected explants in ¼ MS medium supplemented with 93.83 µmol/L of acetosyringone would result in the highest hairy root induction. Whereas the highest transformation could be obtained by inoculation of 7-d-old cotyledons of Bert cultivar with R1000 strain at OD_600_ = 0.32 for 29 min, then incubation of infected explants in 1/2 B5 root induction medium supplemented with 126.74 µmol/L of acetosyringone (**Table 8**). In *ex vitro* experiment, the best fitness function value of hairy root induction was obtained by the interaction of R1000 *A. rhizogenes* strain/line 20SS01/8.45-d-old seedlings, whereas the optimal level of inputs for transformation was R1000 *A. rhizogenes* strain/Bert cultivar/8.66-d-old seedlings (**Table 8**).

**Table 8:** The results of optimization process for *A. rhizogenes*-mediated hairy root transformation of soybean in *in vitro* and *ex vitro* experiments.

Experiment	Fitness function	Optimal level of input variables							Predicted fitness function value
		A. rhizogenes strain	A. rhizogenes cell density (OD_600_)	A. rhizogenes inoculation duration (min)	Plant genotype	Age of explants (d)	Concentrations of acetosyringone (µmol/L)	Root induction media	
*In vitro*	Hairy root induction	R1000	0.38	28.43	20SS01	6.89	93.83	¼ MS	99.54
	*RUBY* gene transformation	R1000	0.32	29.00	Bert	7.00	126.74	½ B5	78.96
*Ex vitro*	Hairy root induction	R1000	–	–	20SS01	8.45	–	–	99.51
	*RUBY* gene transformation	R1000	–	–	Bert	8.66	–	–	59.28

### 3.6 Molecular analysis of transformants

**Figure 5:**
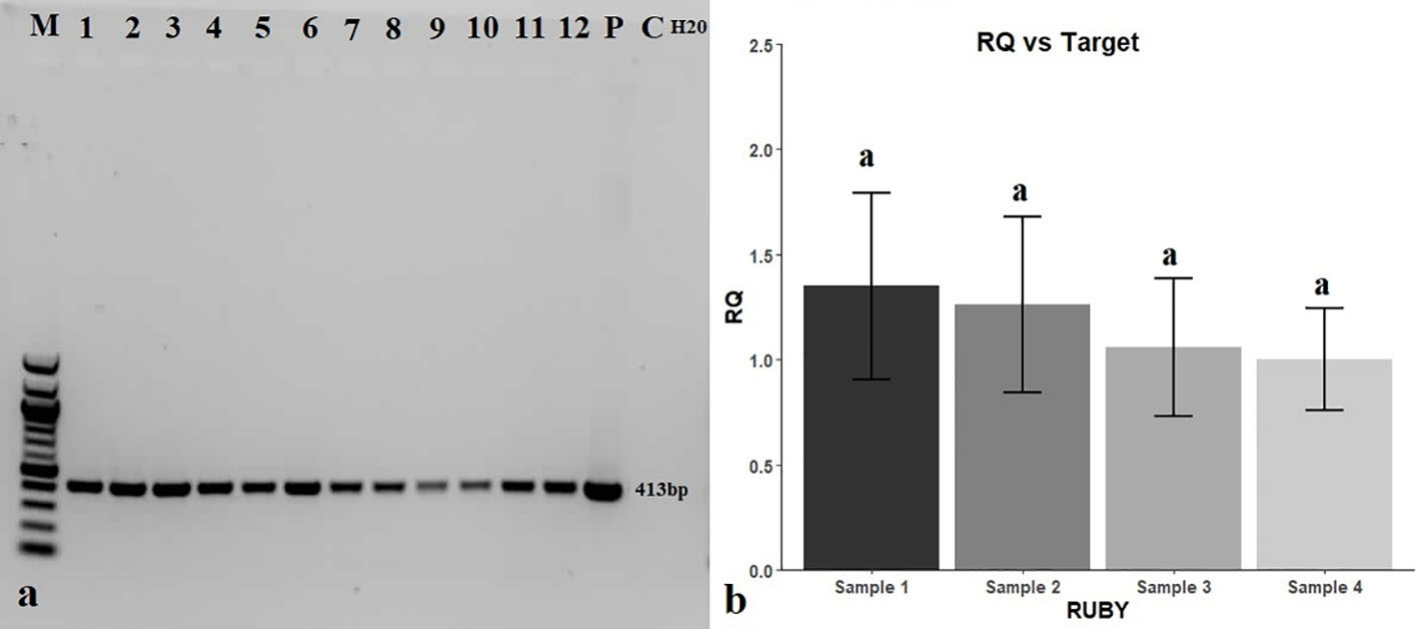
Molecular analysis of putative transgenic red hairy roots for the presence of the *bar* gene and expression of the *RUBY* gene. **(A)** PCR detection of the *bar* gene in transgenic hairy roots. M: DNA marker (100 bp), 1-12: red putatively transgenic hairy roots, P: 35S_Ω_RUBY plasmid as a positive control (+), C: white hairy roots as a negative control, H_2_O: negative control without template. **(B)** Expression of the *RUBY* reporter gene in transgenic hairy roots normalized relative to ubiquitin (Gma.441.1.S1_at) and actin (GmaAffx.90181.1.A1_at) genes using the 2^-△△CT^ method.

Putative transgenic roots (red roots) were examined for the presence of the *bar* gene by PCR amplification. All DNA samples extracted from red roots produced the 413-bp amplification product diagnostic of the *bar* gene, while DNA extracted from white roots (C, for control) did not (**Figure 5A**). PCR amplification of the *rol* C gene and the absence of expected band (520 bp) proved that there is no *A. rhizogenes* contamination in tested transgenic hairy roots (**Figure S4**). Results of the RT-qPCR analysis revealed that the relative expression level of the *RUBY* gene was constant throughout hairy roots as there was no significant difference between the expression levels measured in the initial (sample 1), middle (sample 2), and terminal parts (sample 3) of red hairy roots, compared with the control (sample 4, composite of samples 1-3) (**Figure 5B**).

## 4 Discussion

The *in vitro* approach offers a rapid, simple, and highly efficient system to produce a large mass of transgenic hairy roots in soybean (Chen et al., 2018; Cheng et al., 2021). In addition, fast screening for effectiveness of the prepared expression vectors and testing various factors and components is achievable through *in vitro* hairy root induction (Michno et al., 2015). Factors such as plant genotype, developmental stage of explant, *A. rhizogenes* strain, medium composition, and culture environment affect the efficiency of *in vitro* hairy root induction (Thwe et al., 2016). Other parameters, such as cell density, the time of inoculation, the choice and concentration of antibiotics used for *Agrobacterium* eradification, the selectable agent and marker, and acetosyringone concentration, must be optimized for an efficient *in vitro Agrobacterium*-mediated delivery of gene(s) of interest (Niazian and Niedbała, 2020).

Although K599 is the most common strain used to optimize *in vitro* hairy root induction in soybean (Chen et al., 2018; Cheng et al., 2021; Huang et al., 2022), testing different strains and finding the most effective one is necessary, as hairy root stimulation and gene transfer efficiency are directly influenced by the *A. rhizogenes* strain used (Thwe et al., 2016) and differential efficiencies of *A. rhizogenes* strains have been reported in hairy root induction of other plants such as African geranium (Yousefian et al., 2021), fenugreek (Tariverdizadeh et al., 2021), tobacco (Qin et al., 2022; Yektapour et al., 2022) and marshmallow (Tavassoli and Safipour Afshar, 2018). In this study, the effect of *A. rhizogenes* strains was significant on *in vitro* hairy root induction percentage and transformation efficiency. The R1000 strain proved more efficient than ARqual, A4 and K599. A higher intrinsic capacity of the Ri plasmid and subsequent successful transfer of the T-DNA into the host cells in R1000, compared to other strains, has been previously documented (Thilip et al., 2015; Joseph Sahayarayan et al., 2020).

With the same *A. rhizogenes* strain, the optimum percentage of hairy roots can be influenced by the infection period (inoculation duration) and *A. rhizogenes* cell density (Boobalan and Kamalanathan, 2020). This is despite previous studies optimized *in vitro* hairy root transformation of soybean with a constant level of these parameters (OD_600 = _0.6/30 min) (Chen et al., 2018; Cheng et al., 2021), (OD_600 = _0.8-1/30 min (Huang et al., 2022). In the first *in vitro* experiment of the present study, the effects of different *A. rhizogenes* cell densities and inoculation durations on hairy root transformation were evaluated using an orthogonal experimental design. Although the optimal inoculation duration was the same for all investigated strains (30 min), the optimal cell density varied. Lower cell density leads to better results with the R1000 strain, suggesting that this strain is more aggressive than the other three studied.

Basal root induction media and acetosyringone are two other parameters whose effects on *in vitro* hairy root induction and transformation efficiency were significant in this study. The synergistic effect of the culture media on *in vitro* hairy root induction of soybean has been recently reported by Cheng et al. (2021). In this work, the authors found that ½ B5 was better than MS, B5 and ½ MS media, leading to a transformation efficiency of 53% for the *GUS* reporter gene. However, in the mentioned study, a constant concentration of acetosyringone (40 mg/L) was applied in root induction media (Cheng et al., 2021), while its optimal concentration is known to vary for different plant genotypes (Balasubramanian et al., 2018). In this study, for obtaining optimal conditions, different concentrations of acetosyringone were tested and 150 µmol/L was found to be the optimum concentration, with higher transformation efficiency than in the previously published work (80%). Insufficient and excessive concentrations are thought to lead to failure in reducing *Agrobacterium* virulence and the toxic effect on *Agrobacterium* and plant cells, respectively (de Clercq et al., 2002).

Plant genotype is an important factor that limits the transformation efficiency of soybean (Xu et al., 2022).Different *in vitro* transformation frequencies (58-69%) have been reported in soybean genotypes Williams 82, Zhonghuang 13, Magellan and Maverick when inoculated with strain K599 (Cheng et al., 2021). Chen et al. (2018) have reported different percentages of hairy root induction (90-99%) and transformation efficiency (30-60%) in Williams 82, Jack, Zigongdongdou, Heihe 27 and Zhonghuang 30 cultivars using strain K599. In another study, transformation efficiencies of 62.7% and 70.1%, respectively, have been reported for Williams 82 and Tianlong 1 using strain K599 (Huang et al., 2022). Here, we also tested different plant genotypes, across different *A. rhizogenes* strains, and observed a significant effect of the genotype on *in vitro* hairy root induction and transformation efficiency. Transformation efficiencies obtained with cv. Bert and line 20SS01were not significantly different, which shows that the optimized *in vitro* approach can be effective for both model and specific genotypes.

The age of the plant material is one of the most critical factors that can affect the efficiency of hairy root transformation (Hu and Du, 2006). The effect of explant age has only been investigated in one of the previous *in vitro* optimization studies in soybean, where the authors reported no difference between the cotyledons from 1-, 2-, 3-, 4-, 5-day-germinated seeds as explants in terms of hairy root transformation efficiency (Cheng et al., 2021). Contrary to the previous report, here we observed a significant effect of explant age on hairy root induction and transformation efficiency and 7-d-old explants were better than juvenile and older materials.

The *ex vitro* inoculation (hypocotyl stabbing) is a rapid and highly efficient system of producing soybean composite plants that can be done in two- (Kereszt et al., 2007) or one-step (Tóth et al., 2016; Fan et al., 2020b). The *ex vitro* method is faster and more cost-effective than the *in vitro* technique as aseptic conditions have been eliminated (Niazian et al., 2022). Besides, an *A. rhizogenes* paste, grown in glycerol-containing medium, makes the *ex vitro* technique faster and easier as we do not need to prepare cell cultures at a precise density. Different parameters, such as plant genotype, developmental stage of the explant and bacterial strain can affect the efficiency of the *ex vitro* method.

Similar to the *in vitro* method, the effect of bacterial strain, plant genotype and age of explant on *ex vitro* hairy root induction and transformation efficiency was also significant. K599 is the only *A. rhizogenes* strain to have been used in previous experiments conducted to optimize *ex vitro* hairy root transformation of soybean (Kereszt et al., 2007; Cao et al., 2009). Here, different strains, including R1000, A4, ARqual and K599 were tested on different soybean genotypes and R1000 was found to be the most efficient strain. Although transformation efficiency in this study was lower than that reported by Cao et al. (2009), (60% *vs* 94.2%), these authors used different genotypes (Beifeng 11, Heihe 27, Jindou 19, Suinong 14, Yudou 25, Yuechun 04–5, Zhongdou 19, Zhonghuang 40, Zhongpin 661 and Zigongdongdou) in their optimization protocol.

Significant interactions were observed between the studied parameters in both *in vitro* and *ex vitro* approaches of the present study. *Agrobacterium*–host interactions is the most important factor that could influence the *A. rhizogenes*-mediated transformation efficiency (Colling et al., 2010). Therefore, finding the best combination of *Agrobacterium*-host genotype is essential to achieve a higher transformation rate. Somewhat surprisingly, this interaction had not been investigated in any of the previous optimization studies in soybean (Cao et al., 2009; Cheng et al., 2021; Huang et al., 2022). Here, we found it to be very significant in both *in vitro* and *ex vitro* approaches. The inoculation of cv. Bert with the R1000 strain was the best combination of *Agrobacterium*–host for transformation efficiency.

The combination of machine learning algorithms and optimization algorithms is an efficient method to figure out the complex interactions of inputs and predict optimized combinations of factors for desired outcomes and subsequently reduce the volume of multifactorial studies (Pepe et al., 2021). Hybrid MLP-GA algorithms were successfully used to predict and optimize the percentage of hairy root induction and transformation efficiency under the effects of studied parameters in both *in vitro* and *ex vitro* methods. Suggested optimal level of inputs were near the values obtained from the laboratory experiments showing the efficiency and reliability of machine learning algorithms in the interpretation of *in vitro* and *ex vitro* experiments with multiple-independent variables. To the best of our knowledge, this is the first report of the application of machine learning algorithms for the prediction and optimization of both *in vitro* and *ex vitro* soybean hairy root transformation.

The results of the present study showed faster production of soybean composite plants, with lower transformation efficiency, through the two-step *ex vitro* inoculation compared to the *in vitro* inoculation. Although the *ex vitro* technique is easier and faster than the *in vitro* technique, it may lead to lower levels of interaction between the host cells and the bacteria because of fewer or less intimate contacts (Tavassoli and Safipour Afshar, 2018). The *RUBY* gene was used for the easy detection (visible in the early stages of cell division) of transgenic hairy roots in four soybean genotypes through both *in vitro* and *ex vitro* methods. Different reporter genes, such as *GUS*, *GFP* and *DsRed2*, have been used to detect both *in vitro* and *ex vitro* obtained transgenic hairy roots in soybean (Chen et al., 2018; Fan et al., 2020b; Cheng et al., 2021). Detection of these genes is destructive or requires specific equipment or substrates (Niazian et al., 2022). *AtMyb75/PAP1*, which induced purple/red-colored anthocyanin accumulation, is a visible reporter gene that has been applied in soybean *ex vitro* hairy root transformation (Fan et al., 2020a). Anthocyanin is not a universal visible reporter as there are false-negative regenerates using this reporter (Khidr et al., 2017). Here, the integration of T-DNA was successful in all tested red hairy roots. The expression pattern of the *RUBY* gene was constant throughout the length of red hairy roots. These results confirm that *RUBY* gene can be used as reliable convenient and intuitive reporter to improve the detection of soybean transgenic hairy roots using both *in vitro* and *ex vitro* routes. In *in vitro* gene transformation studies, *RUBY* is a valuable reporter gene as it did not affect callus induction, plant regeneration, development or fertility of *Plukenetia volubilis*, *Nicotiana benthamiana*, and *Arabidopsis* (Yu et al., 2023). In addition to “in root” gene functional studies, exciting opportunities for creating new horticultural value is achievable in breeding of ornamental plants through *RUBY* gene expression and heterologous betalain production (Yuan et al., 2022). Single insertion of transgene is the most optimal mode of integration in a transformation assay. In addition to checking the efficiency of the presented protocol, the Southern blotting test would be helpful to evaluate the correlation between the copy number of transgene and intensity of *RUBY*-induced color in hairy roots.

## 5 Conclusions

The *in vitro* and *ex vitro* hairy root transformation assay are components of plant biotechnology and functional genomics. The *in vitro* technique is suitable for production of transgenic hairy roots and their tracking in a controlled environment. The *ex vitro* technique is more suitable for fast induction of transgenic hairy roots and easy in-root functional analysis. In both *in vitro* and *ex vitro* techniques, abundant red hairy roots were obtained after inoculation with *A. rhizogenes* harboring the *RUBY* visible reporter gene. Visual screening of transgenic hairy roots, from the first emerged cells to developed hairy roots, is now possible using the *RUBY* reporter gene. The transformation efficiency of *in vitro* method was more than that obtained with the *ex vitro* technique (80% vs 60%). However, the *ex vitro* technique was faster than the *in vitro* technique in terms of production of composite plants. The interaction effects of *A. rhizogenes* parameters (cell density and inoculation duration) with plant parameters (plant genotype, age of explant) and culture media parameters (root induction media, acetosyringone concentration) were significant on *in vitro* hairy root induction and transformation efficiency. The interaction effect of *A. rhizogenes* strain with plant parameters (plant genotype, age of explant) was significant on *ex vitro* hairy root induction and delivery of the *RUBY* gene. R1000 was the most promising *A. rhizogenes* strain for both *in vitro* and *ex vitro* delivery of *RUBY* reporter gene in the soybean genotypes used in this work. In both *in vitro* and *ex vitro* experiments, according to the GA optimization, the most responsive soybean genotype was different for the best fitness function values of hairy root induction and transformation of *RUBY* genes, however, the combinations of R1000 strain/7-d-old cotyledons and R1000 strain/8.66-d-old seedlings were the best inputs for gene transformation in *in vitro* and *ex vitro* experiments, respectively. PCR and RT-qPCR molecular analyses proved the efficiency and reliability of the *RUBY* gene as a perfect visual reporter gene to track in-root transgenic events. The optimized protocols are applicable for fast and reliable functional analysis of genes of interest (overexpression, suppression, knockout). In addition to gene functional analysis, optimized *in vitro* protocol would be useful for quickly determining the cleavage efficiency of the designed gRNA(s) in a CRISPR/Cas9 construct. Interaction of *A. rhizogenes* and plant genotype in an important parameter that can affect the efficiency of the procedure in other target plant species.

## Data availability statement

The original contributions presented in the study are included in the article/**Supplementary Material**. Further inquiries can be directed to the corresponding author.

## Author contributions

Conceptualization: MN and DT. Methodology: MN. Software: MN Validation: MN, DT, FB, and SC. Formal analysis: MN. Investigation: MN, MR, and DT. Resources: DT. Data curation: MN. Writing—original draft preparation: MN. Writing—review and editing: DT, FB, and SC. Visualization: MN. Supervision: DT. Project administration: DT. Funding acquisition, DT and FB. All authors have read and agreed to the published version of the manuscript.
