# Supplementary material for: Optimization of in vitro and ex vitro Agrobacterium rhizogenes-mediated hairy root transformation of soybean for visual screening of transformants using RUBY
Source: Front Plant Sci. 2023 Jul 7;14:1207762. doi: 10.3389/fpls.2023.1207762 (PMC10361064; doi:10.3389/fpls.2023.1207762)
Supplement: Supplementary file 1 [file DataSheet_1.docx]

Supplementary Material

# **Supplementary Table 1**. Composition of nutrient solution used to fertilize soybean plants.

| Compound | Concentration of stock solution  (g L^-1^) | Quantity in the final solution  (mg L^-1^) | Volume of stock solution per L in final solution (ml L^-1^) |
| --- | --- | --- | --- |
| KCl | 745.52 | 745.50 | 10 |
| KNO_3_ | 101.10 | 1011.10 | 10 |
| MgSO_4_.7H_2_O | 246.48 | 492.96 | 2 |
| K_2_HPO_4_ | 174.18 | 174.18 | 1 |
| KH_2_PO_4_ | 136.09 | 136.09 | 1 |
| CaCl_2_.2H_2_O | 147.01 | 147.01 | 1 |
| Fe-Sequestrate 330 | 20.00 | 20 | 1 |
| H_3_BO_3_ | 1.00 |  | 1 |
| MnCl_2_.4H_2_O | 1.00 |  | 1 |
| ZnSO_4_.7H_2_O | 0.58 |  | 1 |
| CuSO_4_.5H_2_O | 0.13 |  | 1 |
| Na_2_MoO_4_.2H_2_O | 0.10 |  | 1 |
| CoCl_2_.6H_2_O | 1 ml of solution (1x10-3 g / L) |  | 1 |

# **Supplementary Table 2.** Forward and reverse primers used in qPCR experiment.

| Gene ID | Direction | Sequence (5' to 3') |
| --- | --- | --- |
| Gma.441.1.S1_at | Forward | AGTGTAGATGCCATATCAGAACC |
|  | Reverse | CCACCAATAGTAGCCACAATTAC |
| GmaAffx.90181.1.A1_at | Forward | AAGCACTTCCGATGAACGATAG |
|  | Reverse | CTTGGCATCACTTAGCACCTTC |
| RUBY | Forward | CATGTACCAGTTCAAGTACCC |
|  | Reverse | AGGATCTCTTCCACCTTTCT |
| rol C | Forward | ATGGCTGAAGACGACCTGTGT |
|  | Reverse | TTAGCCGATTGCAAACTTGCA |

# **Supplementary Table 3**. Analysis of variance of hairy root induction and transformation efficiency in soybean line 20SS01 via in vitro transformation. Three parameters (*Agrobacterium rhizogenes* strain, cell density and duration of inoculation) were explored in a factorial experiment where each of 36 treatments (five cotyledons/treatment) was replicated three times.

| Source of variation | df | Mean squares | |
| --- | --- | --- | --- |
|  |  | Hairy root induction | Transformation efficiency |
| Strain | 3 | 5406.17** | 0.44** |
| Cell density (OD600) | 2 | 9048.14** | 0.55** |
| Inoculation duration | 2 | 6459.25** | 0.55** |
| Strain × Cell density | 6 | 4020.98** | 0.25** |
| Strain × Inoculation duration | 6 | 572.83** | 0.09** |
| Cell density × Inoculation duration | 4 | 481.48** | 0.15** |
| Strain × Cell density × Inoculation duration | 12 | 498.76** | 0.15** |
| Error | 72 | 48.14 | 0.02 |
| Coefficient of Variation (%) |  | 25.14 | 19.65 |

** Significant at 1% probability level.

# **Supplementary Table 4**. Analysis of variance of hairy root induction and transformation efficiency in soybean via in vitro transformation. Three parameters (Agrobacterium rhizogenes strain, plant genotype and age of explant) were explored in a factorial experiment where each of 48 treatments (five cotyledons/treatment) was replicated three times.

| Source of variation | df | Mean squares | |
| --- | --- | --- | --- |
|  |  | Hairy root induction | Transformation efficiency |
| Plant genotype | 3 | 1855.55** | 0.34** |
| Strain | 3 | 18166.66** | 1.70** |
| Age of explants | 2 | 23544.44** | 1.81** |
| Plant genotype × Strain | 9 | 1245.67** | 0.15** |
| Plant genotype × Age of explants | 6 | 911.11** | 0.14** |
| Strain × Age of explants | 6 | 1555.55** | 0.44** |
| Plant genotype × Strain × Age of explants | 18 | 216.04** | 0.06** |
| Error | 96 | 83.33 | 0.03 |
| Coefficient of Variation (%) |  | 28.57 | 22.21 |

** Significant at 1% probability level.

# **Supplementary Table 5.** Analysis of variance of hairy root induction and transformation efficiency in soybean cv. Bert using A. rhizogene strain R1000 via in vitro transformation. Two parameters (acetosyringone concentrations and root induction media) were explored in a factorial experiment where each of 12 treatments (five cotyledons/treatment) was replicated three times.

| Source of variation | df | Mean squares | |
| --- | --- | --- | --- |
|  |  | Hairy root induction | Transformation efficiency |
| Concentrations of acetosyringone | 2 | 8711.11 ** | 81.37 ** |
| Root induction media | 3 | 3511.11 ** | 25.17 ** |
| Concentrations of acetosyringone × Root induction media | 6 | 977.77 ** | 10.04 ** |
| Error | 24 | 77.77 | 1.33 |
| Coefficient of Variation (%) |  | 16.19 | 26.87 |

** Significant at 1% probability level.

# **Supplementary Table 6.** Analysis of variance of hairy root induction and transformation efficiency in soybean via ex vitro transformation. Three parameters (Agrobacterium rhizogenes strain, plant genotype and age of explant) were explored in a factorial experiment where each of 48 treatments (five cotyledons/treatment) was replicated three times.

| Source of variation | df | Mean squares | |
| --- | --- | --- | --- |
|  |  | Hairy root induction | Transformation efficiency |
| Strain | 3 | 2736.11** | 1.09** |
| Plant genotype | 3 | 3150.92** | 0.43** |
| Age of explants | 2 | 26108.33** | 4.81 |
| Strain × Plant genotype | 9 | 963.27** | 0.06ns |
| Strain × Age of explants | 6 | 641.66** | 0.22** |
| Plant genotype × Age of explants | 6 | 434.25** | 0.01ns |
| Strain × Plant genotype × Age of explants | 18 | 439.19** | 0.12** |
| Error | 96 | 136.11 | 0.04 |
| Coefficient of Variation (%) |  | 24.77 | 32.15 |

ns: not significant, ** Significant at 1% probability level.


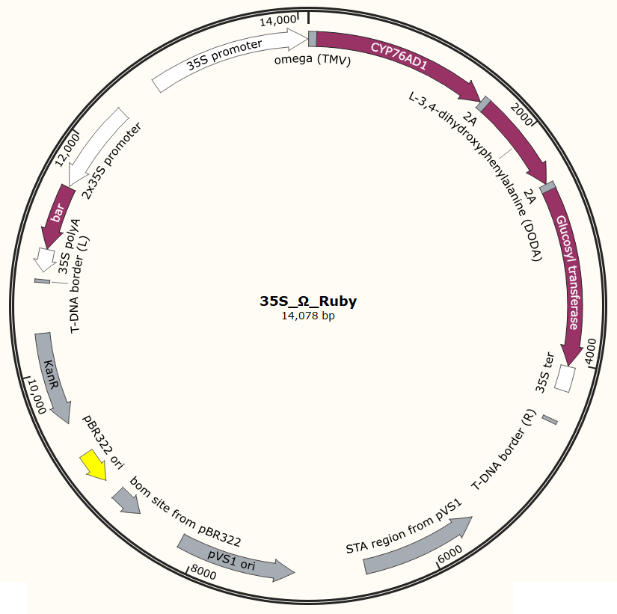


**Fig. S1** The schematic representation of the 35S_Ω_RUBY binary vector used to optimize hairy root transformation of soybean.


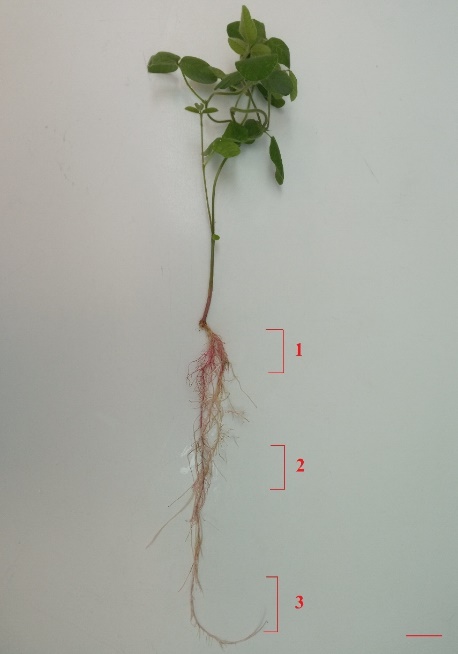


**Fig. S2** Sampling of different parts of transgenic hairy roots to assess the expression pattern of RUBY gene during different developmental stage of hairy roots ((bar= 3 cm).

| 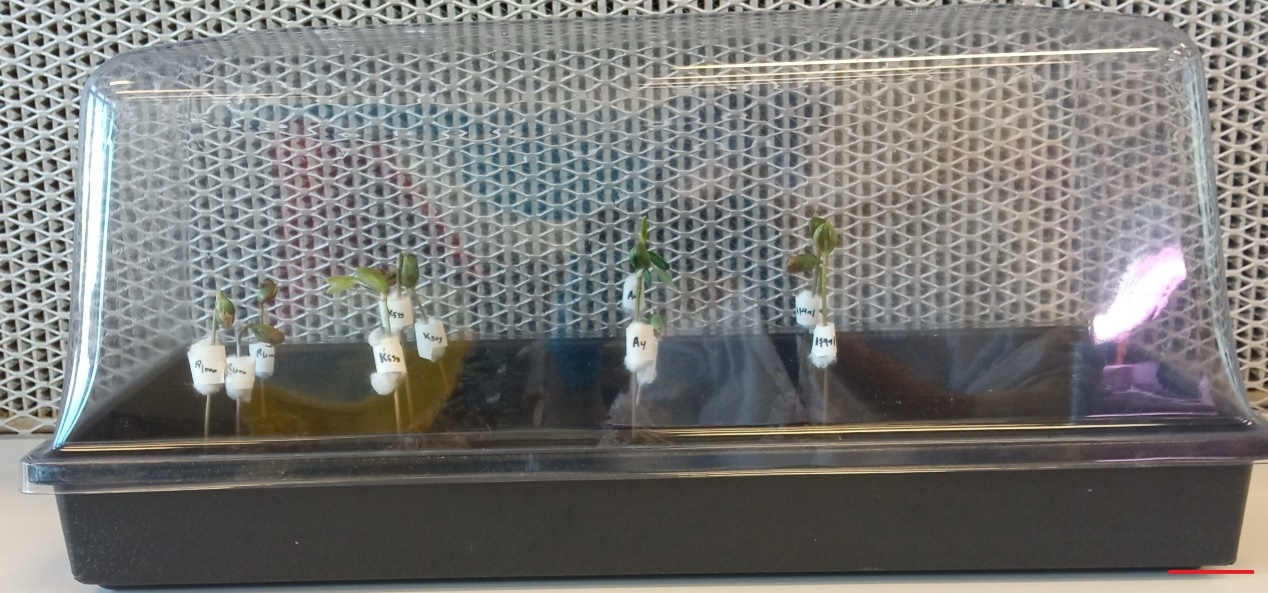 | |
| --- | --- |
| 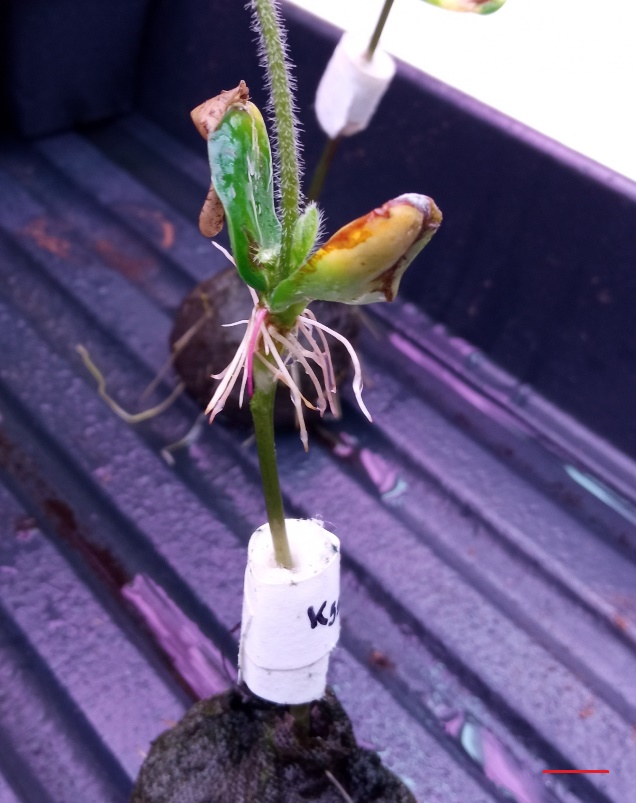 | 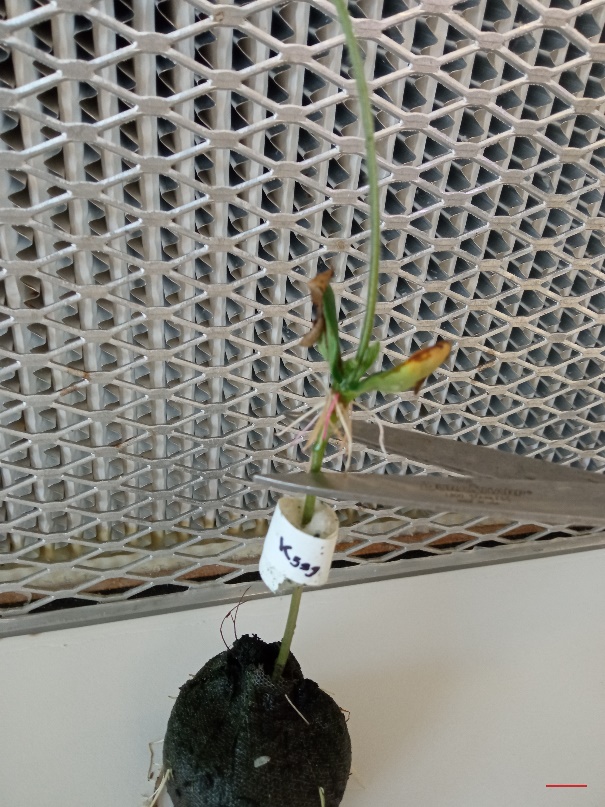 |

**Fig. S3** Hairy root induction and RUBY gene expression in soybean using hypocotyl stabbing technique. (a) Keeping A. rhizogenes-infected soybean plantlets in humid chamber (bar= 5 cm). (b) High mass of white and red hairy roots in the infection sites of hypocotyls (bar= 2 cm). (c) Removal of primary roots by cutting the hypocotyl under the emergence point of hairy roots (bar= 2 cm).

| 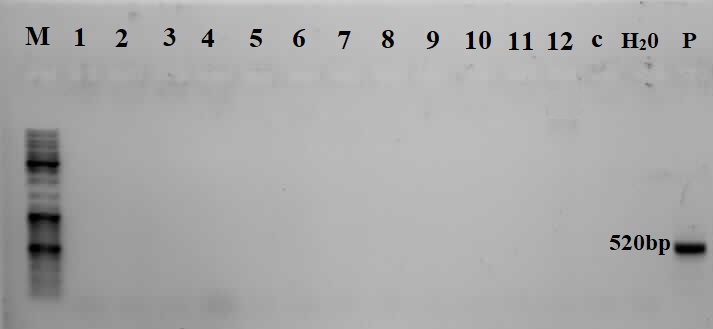 |
| --- |
| **Fig. S4** The PCR amplification of rol C gene (expected size 520 bp) to identify Agrobacterium contamination in putative transgenic hairy roots. M: DNA marker (520 bp marked), 1-12: red putatively transgenic hairy roots, C: white hairy roots as a negative control, H2O: negative control without template, P: Agrobacterium plasmid as a positive control (+). |
